# Associations of built environment features with multimorbidity: A systematic review protocol

**DOI:** 10.1177/26335565251333278

**Published:** 2025-05-05

**Authors:** Alistair L. Carr, Philip Broadbent, Frederick K. Ho, Bhautesh Jani, Jonathan R. Olsen, Valerie Wells, Frances Mair

**Affiliations:** 1 General Practice and Primary Care, School of Health and Wellbeing, 3526 University of Glasgow, Glasgow, UK; 2 MRC/CSO Social and Public Health Sciences Unit, School of Health and Wellbeing, 3526 University of Glasgow, Glasgow, UK; 3 Public Health, School of Health and Wellbeing, 3526 University of Glasgow, Glasgow, UK

**Keywords:** multimorbidity, built environment

## Abstract

**Introduction:**

Preventing or delaying multimorbidity (people living with two or more chronic conditions) is a public health priority. It is currently uncertain if multimorbidity is associated with features of the built environment, a term describing human-made or modified features of the surroundings in which humans live.

**Aims:**

To undertake a systematic review of the literature to determine if built environment features and interventions are associated with multimorbidity and to review the analytical methods used and their implications for causal inference.

**Methods:**

Four databases will be searched (Medline, Embase, Science Citation Index Expanded, and Social Sciences Citation Index) using a prespecified search strategy that incorporates terms for both multimorbidity and the built environment, which includes aspects of neighbourhood design, transport interventions, natural environment, food environments, and housing. Inclusion criteria will include: 1) involves community-based adult populations not selected based on an index condition; and 2) a built environment exposure or intervention was assessed; and 3) outcomes include multimorbidity prevalence, incidence, or trajectory. Reference lists of included studies and previous reviews will also be searched. Two reviewers will independently screen, data extract, and quality appraise (using the ROBINS-E or RoB 2 tool). Results will be synthesised by meta-analysis or, if heterogeneity is too great, according to Synthesis without meta-analysis (SWiM) guidelines. Results will be grouped by type of exposure or intervention and by study quality.

**Conclusions:**

This systematic review will improve understanding of built environment associations with multimorbidity. It could identify aetiological pathways that support the development of multimorbidity-preventative strategies.

## Introduction

Multimorbidity (people living with two or more long-term conditions) is a significant health challenge. Globally, it affects 37% of adults.^1^ The impacts on individuals, their families, and society are significant as it is associated with poorer health, lower quality of life, increased risk of death, and higher healthcare use.^2,3^ Preventing or delaying the development of multimorbidity are research priorities.^4,5^ However, this requires an improved understanding of modifiable risk factors such as the built environment. The built environment describes human-made or modified buildings, infrastructure, and spaces that provide a setting for human activity.^6^ It includes features such as green space, air pollution, and neighbourhood walkability. Changing built environment features could contribute to multimorbidity prevention strategies if they are causally associated with multimorbidity.

Features closely linked to the built environment have been associated with multimorbidity, such as housing tenure, household composition, rurality, area-level deprivation, and social environment features.^7,8^ Furthermore, built environment features have been associated with health behaviours and single conditions. These include diet, physical activity, alcohol use, obesity, depression, respiratory conditions, cardiovascular disease, type 2 diabetes, and some cancers.^9–14^ However, the role that specific built environment features may have in multimorbidity development is uncertain as the current evidence is limited to scoping reviews.^8,15,16^ Conclusions based on these are limited as the search strategies were either not focused on the built environment^8,16^ or were restricted to Canadian studies.^15^ Additionally, being scoping reviews, they did not formally assess the risk of bias and internal validity of individual studies. The scoping reviews identified that many built environment exposures have been analysed in relation to multimorbidity (air pollution, noise pollution, water pollution, walkability, green space, tree cover, neighbourhood problems, food outlet availability, transport infrastructure, and crime).^8,15,16^ Of these, consistent associations were only reported between air pollution and multimorbidity.^8^ The other exposures had only been analysed in relatively few studies or had mixed findings.^8,15,16^ Therefore, there is a knowledge gap about what built environment features other than air pollution are associated with multimorbidity.

The scoping reviews highlighted that improved study methods are needed to support better explanations of causal mechanisms.^8,15,16^ Although randomised controlled trials are the gold standard for assessing causation, observational studies can still strengthen causal inference. This can be determined by interpreting evidence according to the Bradford Hill principles of causation^17^ and by assessing if analytical issues specific to geographical studies have been accounted for.^18,19^ Bias can be introduced to geographical analyses from neighbourhood boundary definitions, residential self-selection (where health behaviour preferences influence residential location), and spatial autocorrelation (where built environment exposures are related to other area-based characteristics based).^18,19^ It is currently unclear how these issues have been addressed in the multimorbidity-built environment literature. Addressing this knowledge gap is important as it could impact what causal inferences can be made from reported associations and highlight future research needs.

We hypothesise that a range of built environment features are associated with multimorbidity incidence, prevalence, and trajectory. This is based on the previously reported associations of built environment features with health behaviours, clinical risk factors and chronic conditions, and of urban-rural status and area-level deprivation with multimorbidity.^7–14^ The primary aim of this systematic review is to determine whether built environment features and interventions are associated with multimorbidity prevalence, incidence, or trajectory in adults. Secondary aims are to determine: 1) what built environment features and interventions have been analysed in relation to multimorbidity prevalence, incidence, or trajectory in adults; 2) what analytical methods have been used to evaluate the associations of built environment features and interventions; and 3) what, if any, are the gaps in evidence and future research needs in this area.

## Methods

This systematic review protocol was registered in PROSPERO (CRD 42024621458) and will be conducted in accordance with the Preferred Reporting Items for Systematic Reviews and Meta-analysis (PRISMA) guidelines.^20^

### Eligibility criteria

The study eligibility criteria are summarised in Table 1.

**Table 1.** Study eligibility criteria.

Feature	Inclusion	Exclusion
Population	Community-dwelling adults (≥16 years) living with or without multimorbidity	Studies in institutionalised care settings such as nursing homes, hospitals, or mental health facilities Participants selected based on the presence of an index health condition or conditions (i.e., studies of comorbidity) Studies where all or most participants were aged less than 16 years
Exposure or intervention	Study exposure(s) included at least one external built environment feature or intervention, where an intervention aimed to create or physically modify a built environment feature or set of features.^a^	Features or interventions of the healthcare-built environment or internal built environment (e.g. inside workplaces or retail premises) Area-level characteristics that do not describe specific built environment characteristics (e.g. urban-rural status and area-level deprivation) Behavioural interventions and interventions that did not result in a physical modification to the built environment
Comparison	No comparison or control group will be required for study inclusion	Not applicable
Outcome	Multimorbidity prevalence, incidence, or trajectory.^b^	Comorbidity outcomes, where an outcome of a specified condition or set of conditions was reported relative to an index condition
Study design	Any peer-reviewed quantitative study design	Qualitative studies, case reports or series, letters, editorials, reviews, and conference abstracts

^a^The built environment describes human-made or human-modified buildings, infrastructure, and spaces that provide a setting for human activity. The built environment scope will include: neighbourhood design (e.g. street connectivity, land use mix, walkability, proximity of local amenities), housing (e.g. density, type, quality), the food environment (e.g. availability of healthy food options, exposure to fast food outlets), the natural environment (e.g. green and blue spaces, air pollution), and transport networks (e.g. public transport provision, traffic calming measures, active travel infrastructure).^21^

^b^Multimorbidity is the occurrence of two or more chronic conditions in the same individual, where none is considered an index condition.^22^

#### Population

Studies will be included that were conducted in populations where all or most participants were adults aged 16 years or over and were conducted in a community setting (i.e. not in a hospital, care home, or prison). Studies that selected their study population based on the presence of an index condition or set of conditions will be excluded. This is to exclude comorbidity studies. Multimorbidity differs from comorbidity as it does not prioritise an index condition or set of conditions.^22^ Multimorbidity studies are, therefore, more representative of the general population living with multiple chronic conditions. Excluding comorbidity studies is in keeping with previous multimorbidity reviews.^8,23^

#### Exposure or intervention

Studies that reported an association with at least one built environment feature or intervention will be included. For this review, the built environment scope will be based on the British Columbia Centre for Disease Control Healthy Built Environment Linkages Toolkit (‘HBE Linkages Toolkit’).^6^ It conceptualises the built environment as consisting of external features related to neighbourhood design, housing, the food environment, natural environments, and transport networks (Table 2). This model was selected as it focuses on modifiable built environment features that are associated with health and can be influenced by public health policy.^6,24^ Features will be included that may have either a positive or negative impact on health. Built environment exposures measured at an individual, household, or community level using objective (e.g., mapping approaches) or subjective (e.g., survey) methods will be included.

**Table 2.** Aspects of the built environment that can influence health.

Built environment aspect	How this aspect can influence health	Examples
Neighbourhood design	A neighbourhood design that supports health is reflected in diverse neighbourhoods where all people can live, work, play, connect, and access amenities	Proximity of amenities and recreational facilities Neighbourhood crime and perceptions of safety Mixed residential and commercial land use Compact urban designs and street connectivity to increase proximity to work, school, recreation, shopping, and other errands Development of vacant or underutilised land
Transportation networks	A transportation network that supports health is safe, affordable, accessible to all levels of mobility, and prioritises active transportation options like walking, cycling, and public transit	Availability of public transport Developing dedicated rail lines and bus lanes Road pricing and high-priced parking Spatially separated pavement and cycle lanes Traffic calming measures
Natural environments	A natural environment that supports health is one in which green spaces and natural elements are protected, incorporated into the built surroundings, and accessible to all people including children, low-income residents and people with chronic conditions or disabilities	Access to natural trails, parks, and other green spaces Trees and vegetation across the built environment Air pollution Ambient air temperature and the health island effect
Food environment	A food system that supports health maintains equitable access to affordable, safe, nutritious, and culturally appropriate foods	Proximity to healthy food retail options Exposure to fast food outlets Farmers markets
Housing	Housing that supports health protects people from health hazards inside and near the home and is safe, affordable, and accessible to all	Diversity of types of housing and tenure Access to good quality housing (e.g., energy efficient homes) Housing sited away from busy roads or industrial sites to minimise exposure to environmental hazards (e.g., noise and radon exposure)

Source: British Columbia Centre for Disease Control Healthy Built Environment Linkages Toolkit: making the links between design, planning and health.^6^

It is necessary to limit the scope of the built environment as its broad definition could incorporate all human-made or human-modified spaces, buildings, and infrastructure. This could include internal building environments (e.g., inside offices, shops, and transport terminals), healthcare facilities, water and energy infrastructure, and broader aspects of the natural environment, such as biodiversity and climate change. This review will exclude these features to focus on the modifiable built environment that public health policies can most readily target. Area-level deprivation and urban-rural status will also be excluded as they do not describe specific features of the built environment.

Studies of built environment interventions will also be included. These will be defined as interventions that aimed to create or physically modify a built environment feature or set of features, regardless of whether they aimed to improve health. This will include:

1. Interventions that improved the quality of the existing built environment (e.g., development of underutilised or derelict land, street lighting)
2. Interventions that created new built environment features or facilities (e.g. new cycle lane, leisure centre, or playpark)
3. Interventions that restricted access to or reduced exposure to potentially harmful built environment features (e.g. noise restrictions, fast food outlet school exclusion zones)
4. Transport interventions (e.g. speed limits, congestion charging, low emission zones).

Interventions that aimed to increase awareness or usage of existing built environment features or facilities (e.g. improved signage, publicity campaigns, extended opening hours) and behavioural change interventions will be excluded. This would include social prescribing interventions for clinical populations such as green gyms, exercise referral schemes, nature-based walking groups, and gardening groups. Multi-component interventions that involved a physical change to the built environment in combination with another aspect (e.g. new cycle lanes in combination with personalised travel planning and provision of maps) would only be included if associations attributable to the built environment component were reported separately.

#### Comparator

Although a comparison or control group would be preferred, it will not be required for study inclusion.

#### Outcome

Studies will be included that reported multimorbidity incidence, prevalence, or trajectory as outcomes. Multimorbidity will be defined as the occurrence of two or more chronic conditions in the same individual where none of the conditions were considered index conditions.^5,22^ Multimorbidity trajectory will be defined as a change in complexity or number of chronic conditions in people living with multimorbidity. Studies will be included that assessed multimorbidity status using any assessment method (i.e. healthcare records, clinical assessment, prescriptions, self-report, or a validated questionnaire). The chronic conditions that could potentially contribute to the assessment of multimorbidity status will be based on the inclusion of two or more of the 35 conditions listed in the Ho et al. Delphi consensus study^25^ or the 40 conditions developed by Barnett et al. for use in epidemiological analyses^26,27^ (Supplemental Table 1). Comorbidity studies that only report the incidence or prevalence of multimorbidity in the context of a single specified index condition will be excluded.

Secondary outcomes will include any additional multimorbidity health outcomes that were reported. This would consist of any outcomes listed in the Core Outcome Set for Multimorbidity Research consensus study and includes health-related quality of life, mortality, treatment burden, self-rated health, activities of daily living, physical function, and health care costs.^28^

#### Study type

Any quantitative primary study will be included. Preliminary searches suggest that most studies are likely to be observational. These would include ecological, cross-sectional, case-control, cohort, and natural experiment study designs. Experimental study designs would also be included. These would include non-randomised controlled trials, cluster randomised controlled trials, and randomised controlled trials. Qualitative studies, editorials, systematic or non-systematic reviews, letters, conference abstracts, and case studies or reports would be excluded.

### Search strategy

Four databases will be searched: Medline (accessed via Ovid), Embase (accessed via Ovid), Science Citation Index Expanded (accessed via Web of Science), and the Social Sciences Citation Index (accessed via Web of Science). These were selected to include general medical and sociological databases. In addition, we will screen reference lists from included studies and relevant reviews. The Medline search strategy was developed with an Information Scientist (Table 3). It consists of searching for (1) multimorbidity terms, (2) built environment terms, and terms relating to the built environment aspects of (3) neighbourhood design, (4) food environment, (5) natural environment, (6) housing, and (7) transport networks.^6^ These terms will then be combined [(1) and (2 or 3 or 4 or 5 or 6 or 7)]. There will be no restrictions by publication date or country. Grey literature will not be searched and only English-language studies will be included.

**Table 3.** Medline search strategy.

	Search terms
Multimorbidity search terms	
1	Multimorbidity/
2	multi-morbid*.ti,ab
3	multimorbid*.ti,ab
4	co-morbid*.ti,ab
5	comorbid*.ti,ab
6	(multipl* adj2 (long-term or long term or chronic) adj2 (condition* or disease* or disorder* or illness*)).ti,ab
7	or/1-6
Built environment search terms	
8	Built Environment/or Environment Design/
9	((Built or physical or urban) adj1 environment*).ti,ab
10	((feature* or character* or design* or surrounding* or layout* or infrastructure) adj2 (spatial or physical or urban)).ti,ab
11	or/8-10
Neighbourhood design search terms	
12	((feature* or character* or facilit*3 or amenit* or layout* or infrastructure or planning or sprawl or density or connectivity) adj1 (neigbo?rhood* or community or urban)).ti,ab
13	((cycl* or bike* or bicycle* or pedestrian* or walking) adj1 (network*1 or access* or lane*1 or route*1)).ti,ab
14	(walkable or walkability).ti,ab
15	((area level or area-level) adj2 (domain* or determinant* or correlate*)).ti,ab
16	((Crim* or safety) adj1 (neighborhood* or neighbourhood* or communit* or residen* or urban or road)).ti,ab
17	or/12-16
Food environment search terms	
18	(food* adj2 (environment* or access* or availab* or retail* or outlet* or expos*)).ti,ab
19	(foodscape* or “food desert”).ti,ab
20	or/18-19
Natural environment search terms	
21	Environmental Pollution/
22	((environment*or landscape* or location* or space*) adj1 (rural or natural or open)).ti,ab
23	(“playing field” or wood*1 or woodland or park*1 or parkland or garden*1 or allotment* or river*1 or lake*1 or waterway* or wetland* or “green space*” or greenery or “blue space*”).ti,ab
24	((Air or atmospher*) adj1 (pollut* or emission*)).ti,ab
25	“particulate matter”.ti,ab
26	or/21-25
Housing search terms	
27	(environment* adj1 (hous* or home or living)).ti,ab
28	((feature* or character* or design* or location* or layout* or determinant* or correlate* or mix or tenure* or densit* or availab* or affordab* or type or quality) adj2 (hous* or home)).ti,ab
29	or/27-28
Transport network search terms	
30	((transport* or commut* or travel*) adj1 (active or system* or network* or mode*1 or modalit* or route*1 or connectivity or environment* or infrastructure)).ti,ab
31	(transport* adj1 (public or private)).ti,ab
32	((congestion or traffic or emission) adj1 (charge* or zone*)).ti,ab
33	((speed or traffic or vehicle) adj1 (restriction* or limit* or calming)).ti,ab
34	((noise or sound) adj1 (exposure* or pollution or level* or disturbance* or reduction*)).ti,ab
35	or/30-34
Multimorbidity search terms and (built environment or neighbourhood design or food environment or natural environment or housing or transport network search terms)	
36	7 and (11 or 17 or 20 or 26 or 29 or 35)

### Data screening and extraction

Records will be imported to DistillerSR and duplicates will be removed. Titles and abstracts will be screened independently by two reviewers to identify records that potentially meet the eligibility criteria. Full-text reports will then be retrieved and screened independently by two reviewers. The recorded exclusion reason will follow the hierarchy in Supplemental Table 2 if more than one exclusion reason could apply. Any disputes during screening will be resolved initially by discussion, followed by arbitration by a third reviewer.

Two reviewers will independently extract data using a standardised form. The form will be piloted before application and include the information shown in Supplemental Table 3. It will consist of information about the study population, study type, exposure, outcome, analytical methods, main findings, and whether the biases and limitations of the analysis were discussed. Any disputes in data extraction will be resolved initially by discussion and, if required, arbitration with a third reviewer.

### Quality assessment

The tool used to assess risk of bias will depend on the study type. Observational studies will be assessed using the Risk of Bias in Non-randomised Studies – of Exposure (ROBINS-E) tool^29^ and experimental studies using the Revised Cochrane Risk-of-bias Tool for Randomized Trials (RoB 2) tool.^30^ The ROBINS-E tool evaluates the risk of bias across seven domains (confounding, exposure measurement, participant selection, post-exposure interventions, missing data, outcome measurement, and selection of the reported results). An overall risk of bias judgement is made based on the judgements for each domain (ranging from low to very high risk of bias).^29^ The RoB 2 tool can be used for individually randomised trials, parallel-group trials, cluster-randomised trials, and crossover trials.^30^ As with ROBINS-E, the overall risk of bias judgment is determined by domain-level judgements. Five domains of potential bias are assessed for individual randomised trials (the randomisation process, deviation from intended interventions, missing outcome data, outcome measurement, and selection of reported results) and an additional domain for cluster-randomised trials (bias arising from identification or recruitment of individual participants within clusters).^30^ Two reviewers will independently make risk of bias judgements with disputes resolved initially by discussion and then by arbitration by a third reviewer.

### Data synthesis

The data synthesis methods will be determined after assessing the risk of bias, reported outcomes, number of identified studies, and statistical heterogeneity among studies that analysed the same exposure. Based on these factors, results will be synthesised with a fixed-effects meta-analysis, a random-effects meta-analysis, or according to the Synthesis without meta-analysis (SWiM) framework.^31^ If different outcomes are reported, they will be transformed where possible and statistical heterogeneity will be assessed using the I^2^ statistic.

Based on scoping reviews and preliminary searches, it is anticipated that heterogeneity will be too great to allow meta-analyses.^8,16^ In this case, associations will be analysed and reported following the SWiM guidelines.^31^ Results will be grouped by the type of exposure or intervention assessed and by risk of bias. An effect direction plot will provide a visual representation of the direction of reported effects. If the data allows, subgroup analyses will be based on the exposure assessment method, multimorbidity definition, study population, study setting, and study design.

In addition, a narrative review of the extracted data will be undertaken to answer the secondary research questions on what built environment features have been analysed and what analytical methods have been used. Together, these will inform what additional research is needed to advance the multimorbidity-built environment literature. The built environment features analysed in the identified studies will be compared with the aspects described in the HBE Linkages Toolkit (Table 2).^6^ The analytical methods used will be grouped according to the Bradford Hill principles of causation.^17^ These criteria were selected due to their relevance to epidemiological studies.

#### Patient and public involvement

This review is being completed as part of a doctoral fellowship. The National Institute for Health and Care Research (NIHR) Cambridge University Hospitals Patient and Public Involvement Panel reviewed an overview of the fellowship that included details of this review. Panel comments were supportive of this being an important research area.

## Discussion

This protocol describes a systematic review that will improve understanding of if and how features of the built environment are related to multimorbidity. It will answer questions about the features that have been assessed, associations reported, methods, and identify research gaps. These questions are important as multimorbidity has significant individual and societal impacts.^2,3^ Greater insight about the role, if any, that built environment features have on multimorbidity development could contribute to preventative strategies that modify built environment features. It may also identify aetiological pathways and contribute to risk prediction tools.

### What this adds to the literature

This review will advance the field by using a comprehensive search strategy that is focused on the built environment and by using a systematic approach to assess risk of bias and synthesise evidence. Built environment features have been associated with behaviours and chronic conditions.^9–14^ However, evidence of associations with multimorbidity is limited.^8,15,16^ Scoping reviews have identified studies that analysed a range of built environment features, including walkability, air pollution, and green spaces.^8,15,16^ Air pollution was the only built environment feature that was consistently associated with multimorbidity.^8^ However, two of these reviews were not focused on the built environment.^8,16^ They used broader search strategies and eligibility criteria that additionally included social environmental features, area-level deprivation, and urban-rural status. Another was focused on the built environment but included any health outcome and was restricted to Canadian studies.^15^ Being scoping reviews, they also did not assess study quality or have a systematic approach to evidence synthesis. To our knowledge, the review described here is the first systematic review on this topic.

This review will also advance the field by assessing the methodological, analytical and conceptual issues relevant to causal inference. This is needed so the potential implications of the current evidence for policymakers can be better understood. The scoping reviews identified that there were limitations in the commonly used study methods (mostly based on cross-sectional analyses).^8,15,16^ Observation studies, which are likely to continue to form the majority of studies, can support causal interpretations of associations if these methodological issues are carefully addressed.^18,19,32–34^ Issues of particular importance to the field are how exposure to the built environment features is measured, how neighbourhoods are conceptualised, and if biases related to residential self-selection and autocorrelation of built environment features are mitigated.^18,19^ Residential self-selection describes the concept of health behaviour preferences influencing residential location and spatial autocorrelation describes the correlation of built environment features with other area-based characteristics based on geographical location.^18,19^ If unaccounted for, these issues can introduce confounding and the potential for associations to be falsely reported. To our knowledge, this review will be the first to assess how these issues have been handled. Addressing this will inform the interpretation of reported associations and highlight future research needs.

### Strengths

A strength of this review is that it contains a comprehensive built environment search strategy and eligibility criteria that are based on multiple built environment aspects. These came from an evidence-based model that has previously been used by public health organisations.^6,24^ An additional strength is the explicit multimorbidity eligibility criteria and how this will be differentiated from comorbidity, a common issue in multimorbidity research.^22^ Further strengths are including any quantitative study design and multimorbidity outcome (incidence, prevalence, trajectory), and independent data extraction and quality appraisal using detailed risk-of-bias assessment tools.^29,30^

### Limitations

Variability in built environment exposure and multimorbidity assessment methods will likely limit this review. Built environment exposure could be assessed objectively using mapping approaches that create buffers centred on households or commuting routes or subjectively using survey questions about places visited. Multimorbidity measurement may differ in terms of the conditions that were assessed and the thresholds used to define multimorbidity (e.g. ≥2 conditions, ≥3 conditions, complex multimorbidity involving multiple body systems), common challenges for multimorbidity research.^35^ Further limitations are that the exposure eligibility criteria exclude social environment characteristics. This is a consequence of having a focus on the built environment. However, the built and social environments closely interact, so excluding the social environment will potentially underestimate the role that built environment features have. Area-level deprivation and urban-rural status will also be excluded. Although these area-level characteristics do not describe specific built environment features, they provide important contextual information about socio-economic position and population density. Extracted data will record these characteristics and how they were incorporated into the analysis. Additional limitations are that grey literature will not be searched and non-English language studies will not be eligible, potentially introducing selection bias. The impact will be assessed by recording how many non-English language studies are excluded.

### Potential impact

Strengthening the evidence base for if and to what extent built environment features are associated with multimorbidity will be of interest to policymakers. Accelerating multimorbidity research and aiming to delay disease to the greatest possible extent were research recommendations of the 2023 Chief Medical Officer’s annual report on health in an ageing society.^36^ Similar calls were made in a 2018 Academy of Medical Sciences report, which additionally highlighted evidence gaps in modifiable risk factors that can be the target of prevention strategies.^5^ If multimorbidity and built environment associations are established that are likely to be causal, modifying them could form the basis of population-wide interventions that prevent or delay the onset of multimorbidity. Such strategies can potentially have a larger overall effect than individual-level interventions as they can reduce multimorbidity risk for everyone in a population, regardless of their level of risk.^4^

Although some built environment features are relatively static, such as the location of major roads or parks, many built environment features are highly modifiable and could be targeted by interventions. For example, improving cycle lanes and pavements, introducing traffic-calming measures, changing planning requirements for new housing developments, and creating fast-food exclusion zones. Many of these interventions would likely have environmental co-benefits. Initiatives such as 20-minute neighbourhoods and the WHO European Healthy Cities Network demonstrate that policymakers are interested in modifying the built environment to improve health and environmental sustainability.^37,38^ However, these strategies can require high upfront financial costs and political and public support. Controversy over 20 mph speed limits in Wales and extending the London low-emission zone highlight the potential challenge.^39,40^ Therefore, it is important to develop a robust evidence base that can inform these policy decisions by identifying all potential health benefits, including those relating to multimorbidity.

### Conclusion

Multimorbidity is one of our ageing society’s key health challenges. The built environment is a potential upstream determinant that could be a target of population-wide interventions. At present, relatively little is known about the built environment features associated with multimorbidity and the analytical methods used to establish those associations. This protocol describes a systematic review that will substantially contribute to our understanding of associations between built environment features and multimorbidity. This could inform future multimorbidity prevention strategies and highlight research gaps.

## Supplemental Material

Supplemental Material - Associations of built environment features with multimorbidity: A systematic review protocol Supplemental Material for Associations of built environment features with multimorbidity: A systematic review protocol by Alistair Carr, Philip Broadbent, Frederick Ho, Bhautesh Jani, Jonathan R Olsen, Valerie Wells, and Frances Mair in Journal of Multimorbidity and Comorbidity

## Data Availability

Template data collection forms, data extracted from included studies, data used for all analyses, and analytic code will be available in the published systematic review, as online supplementary material, or in Github. *
