# Supplementary material for: Associations of built environment features with multimorbidity: A systematic review protocol
Source: J Multimorb Comorb. 2025 May 5;15:26335565251333278. doi: 10.1177/26335565251333278 (PMC12053215; doi:10.1177/26335565251333278)
Supplement: Supplemental Material - Associations of built environment features with multimorbidity: A systematic review protocol [file sj-pdf-1-cob-10.1177_26335565251333278.pdf]

Supplementary Tables

Supplementary Table 1. Included conditions that could contribute to the assessment of multimorbidity

Condition	Barnett et al. list	Ho et al. list
Hypertension (treated and untreated)	X	X
Depression	X	X
Painful condition / chronic primary pain	X	X
Asthma (currently treated)	X	X
Coronary heart disease	X	X
Treated dyspepsia / peptic ulcer	X	X
Diabetes	X	X
Thyroid disorder	X	X
Rheumatoid arthritis, other inflammatory polyarthropathies & systematic connective tissue disorders / osteoarthritis	X	X
Hearing loss / hearing impairment that cannot be corrected	X	X
Chronic obstructive pulmonary disease	X	X
Anxiety & other neurotic, stress related & somatoform disorders	X	X
Irritable bowel syndrome	X	
New diagnosis of cancer in last five years / solid organ cancer / heamatological cancers / metastatic cancers / melanoma / benign cerebral tumours that can cause disability	X	X
Alcohol problems / misuse	X	X
Other psychoactive substance misuse	X	X
Treated constipation	X	
Stroke & transient ischaemic attack	X	X
Chronic kidney disease / end stage kidney disease	X	X
Diverticular disease of intestine	X	
Atrial fibrillation / arhythmia	X	X
Peripheral vascular disease	X	X
Heart failure	X	X
Prostate disorders	X	
Glaucoma	X	
Epilepsy (currently treated)	X	X
Dementia	X	X
Schizophrenia (and related non-organic psychosis) or bipolar disorder	X	X
Psoriasis or eczema	X	
Inflammatory bowel disease	X	X
Migraine	X	
Blindness & low vision / vision impairment that cannot be corrected	X	X
Chronic sinusitis	X	
Learning disability	X	
Anorexia or bulimia / eating disorder	X	X
Bronchiectasis	X	X
Parkinson's disease	X	X
Multiple sclerosis	X	X

Viral Hepatitis	X	
Chronic liver disease	X	X
Heart valve disorders		X
Venous thromboembolic disease		X
Aneurysm		X
Addison's disease		X
Cystic fibrosis		X
Paralysis		X
Peripheral neuropathy		X
Autism		X
Post-traumatic stress disorder		X
Long term musculoskeletal problems due to injury		X
Osteoporosis		X
Gout		X
Chronic pancreatic disease		X
Endometriosis		X
Chronic urinary tract infection		X
Anaemia (including pernicious anaemia, sickle cell anaemia)		X
Meniere's disease		X
HIV/AIDS		X
Chronic Lyme disease		X
Tuberculosis		X
Post-acute covid-19		X
Congenital disease and chromosomal abnormalities		X

Source: Epidemiology of multimorbidity and implications for health care, research, and medical education: a cross-sectional study (1) and Measuring multimorbidity in research: Delphi consensus study (2).

Supplementary Table 2. Hierarchy of exclusion reasons if more than one exclusion reason could apply

1. English language
2. Outcomes included multimorbidity incidence, prevalence, or trajectory
3. Study population had no index health condition
4. Community-based study population
5. Adult population
6. Exposure was a built environment characteristic or intervention
7. Observation or experimental study design

Supplementary Table 3. Extraction form headings

Study details
- Author
- Year
- Title

Study population
- Age group
- Mean age, years
- Proportion female
- Ethnicity
- Country
- Setting (e.g. general population, occupational cohort)
- Sample size
- Socio-demographic characteristics of population and area (e.g. socio-economic position, area-based deprivation, urban-rural status)

Study type
- Data source
- Design (e.g. cross-sectional, longitudinal)
- Intervention
- Study duration

Exposure
- Description
- Measure (objective or subjective)
- Level of exposure assessment (e.g. individual, household, or neighbourhood)
- Neighbourhood or place definition
- Method of assessment

Outcome
- Multimorbidity incidence, prevalence, or trajectory
- Multimorbidity definition
- Method of ascertainment (e.g. health records, self-report)
- Secondary outcomes

Analytical methods
- Research question(s)
- Statistical methods used (e.g. regression, correlation, cluster analysis)
- Reporting statistic
- Confounders adjusted for
- Sensitivity analysis conducted
- Methods to deal with missing data
- Biases and limitations of analytical methods discussed
- Use of Patient and Public Involvement and Engagement

Main findings
- Association with multimorbidity reported (yes or no)
- Size of association (e.g. prevalence, OR, HR)
- Multimorbidity prevalence (in the whole population and by subgroup)

References

1. Barnett K, Mercer SW, Norbury M, Watt G, Wyke S, Guthrie B. Epidemiology of multimorbidity and implications for health care, research, and medical education: a cross-sectional study. The Lancet. 2012;380(9836):37-43.
2. Iris SSH, Amaya A-L, Ashley A, Jim D, Kamlesh K, Umesh TK, et al. Measuring multimorbidity in research: Delphi consensus study. BMJ Medicine. 2022;1(1):e000247.
